# Improving the Quality Characteristics and Shelf Life of Meat and Growth Performance in Goose Fed Diets Supplemented with Vitamin E

**DOI:** 10.3390/foods9060798

**Published:** 2020-06-17

**Authors:** Zabihollah Nemati, Kazem Alirezalu, Maghsoud Besharati, Saeid Amirdahri, Daniel Franco, José M. Lorenzo

**Affiliations:** 1 Department of Animal Science, Ahar Faculty of Agriculture and Natural Resources, University of Tabriz, Tabriz 5166616471, Iran; znemati@tabrizu.ac.ir (Z.N.); m_besharati@hotmail.com (M.B.); s.amirdahri60@gmail.com (S.A.); 2 Department of Food Science and Technology, Ahar Faculty of Agriculture and Natural Resources, University of Tabriz, Tabriz 5166616471, Iran; kazem.alirezalu@yahoo.com; 3 Centro Tecnológico de la Carne de Galicia, Parque Tecnológico de Galicia, rúa Galicia n° 4, San Cibrao das Viñas, 32900 Ourense, Spain; jmlorenzo@ceteca.net; 4 Área de Tecnología de los Alimentos, Facultad de Ciencias de Ourense, Universidad de Vigo, 32004 Ourense, Spain

**Keywords:** vitamin E, immune system, growth performance, carcass quality, TBARS, fatty acid

## Abstract

The present study was carried out to investigate the effect of dietary vitamin E on growth performance, cellular immunity, carcass characteristics, and meat quality in geese. Sixty-four one-day-old male geese were selected from 1200 goose chicks with the same average body weight (92.5 ± 2.5 g) and subjected to two treatments (basal diet or control and basal diet plus 120 mg/kg vitamin E supplement) with 4 replicates (8 geese per replicate) for 8 weeks. After slaughter, goose meat was aerobically packed in polyethylene packages and stored at 4 °C for 9 days. The results showed that vitamin E supplementation improved the growth performance, carcass yield percentage, and immune response of goose (*p* < 0.05). The addition of vitamin E in the diet significantly increased the protein and fat content of goose meat but decreased the moisture and ash content with respect to those obtained from the control diet. During storage, meat from the vitamin E treatment showed higher phenolic content and lower thiobarbituric acid reactive substances (TBARSs) and total volatile nitrogen (TVB-N) values than those from the control treatment. Vitamin E supplementation increased the saturated fatty acids (SFAs), monounsaturated fatty acids (MUFAs), and polyunsaturated fatty acids (PUFAs) in goose meat. However, goose meat supplemented with vitamin E displayed a significantly (*p* < 0.05) higher PUFA/SFA ratio than those of the control group. Based on the results, it was concluded that vitamin E could be used to improve the growth performance of goose, the meat composition in terms of the protein and fat content, the nutritional value in terms of the fatty acid composition, and the shelf life.

## 1. Introduction

The ability to use high-fiber feeds, ease of rearing due to animal behavior patterns, and production of valuable by-products, such as feathers and foie gras (fatty liver), are important characteristics of goose breeding that make it suitable for a sustainable agriculture system. However, the production of fatty livers is considered as a cruel practice and is forbidden in many countries, i.e., in EU countries except France, because it is a registered regional and traditional French product. Goose meat consumption is limited compared to other poultry species, especially in European countries and North America, but there is an increasing interest in this type of meat worldwide, as goose meat production increased from 0.474 to 2.3 billion tons in China between 1990 to 2009 [1]. 

Nowadays, the main goal of poultry producers is not only to achieve the highest economic efficiency but also to produce healthy and high-quality food products. The crossbreeding geese used in goose meat production in the world provide a high protein content, low lipid content, and high nutritional quality [2], with high levels of polyunsaturated fatty acids (PUFAs). PUFAs are more susceptible to lipid oxidation compared to saturated fatty acids (SFAs), although this susceptibility also depends on the presence of pro-oxidants and antioxidants [3,4]. The free radicals and metabolites generated from PUFA oxidation often bring adverse effects to animal performance and meat by affecting the immune system as well as the nutritional values and food shelf life [5]. 

During cellular metabolism, free radicals are produced, causing oxidative stress. Indeed, some disorders, such as high environmental temperature, health challenges, and low nutritional quality diets, increase the production of free radicals [6,7,8,9]. The excessive levels of free radicals negatively effects the body’s defense system [7], and may damage the normal function of DNA, phospholipids, and proteins [10,11,12]. 

Feeding geese with an antioxidant-supplemented diet is a simple strategy to improve their oxidative status and consequently the meat quality. Vitamin E is accumulated in cell membranes and acts as an antioxidant, increasing oxidative muscle stability [13]. It has been demonstrated that the addition of vitamin E to animal diets can increase meat shelf life due to less oxidation [14,15]. Deficiency of vitamin E causes a wide variety of disorders in poultry species. This vitamin, together with the glutathione peroxidase system, protects cells against oxidative damage caused by free radicals. Indeed, recent studies have shown that poultry diet supplementation with vitamin E leads to enhanced growth performance [16,17], immune function [18], serum lipid profile [19], fatty acid composition [20], quality of fatty goose liver and chemical composition of breast muscle [21], and shelf life of poultry meat [22]. Several studies showed that the combination of vitamin E and organic Se in diets positively influenced the final body weight of White Koluda goose [23] and shelf life stability and nutritional aspects of goose liver [24], but no differences were observed in the fat content or fatty acid profiles of goose meat.

However, studies on the role of vitamin E in goose nutrition and its fresh or stored meat quality are scarce compared to other farm animals or poultry species. Therefore, the objective of the present study was to determine the effect of diet vitamin E supplementation on growth performance parameters, carcass characteristics, cellular immune system, serum metabolites, and shelf life of goose meat.

## 2. Materials and Methods 

### 2.1. Experimental Design, Bird Management and Diets, and Sample Collection

The study was conducted using Azerbaijan local geese, according to animal care guidelines and all used procedures were approved by Research Bioethics Committee of the University of Tabriz. Sixty-four one-day-old male geese were selected from 1200 chicks and randomly assigned in a completely randomized design with 2 treatments and 4 replicates of 8 geese in each, in a way that all cages had a similar average body weight (92 ± 5.2 g). The geese were raised on floor pens (1.5 × 2.0 m; 6 geese/m^2^), maintained on 23 h of continuous light with 1h of dark, with free access to water and feed. The experiment was conducted from 1 to 8 weeks of age. One chick group treatment received a control diet, which was formulated to meet the 1- to 28- and 29- to 56-day-old nutrient requirements, as proposed by National Research Council (NRC), 1994 (Table 1), and the other group received the same diet supplemented with 120 mg/kg vitamin E. Vitamin E with 98% purity was supplied by Laboratory Siance Iran Co.

### 2.2. Performance Traits

Body weight (BW) and feed intake (FI) were recorded weekly (after 8 h of fasting to remove the feed residual from gastrointestinal tract), and the feed conversion ratio (FCR) calculated as the unit of eaten feed per unit of weight gain (WG). The rearing period (56 days) was divided into four phases (each phase consisted of 2 weeks) and the performance parameters were explained based on the rearing phase. The European poultry efficiency factor (EPEF) was calculated according to the formula:

Viability, % = 100 − Mortality, %

(1) $$\mathrm{EPEF}=\frac{\mathrm{BW}\;\left(\mathrm{kg}\right)\times \mathrm{Viability}\;\left(\%\right)}{\mathrm{rearing}\;\mathrm{period}\;\left(\mathrm{day}\right)\times \mathrm{FCR}}\times 100.$$

### 2.3. Cellular Immunity 

To evaluate a cutaneous basophil hypersensitivity (CBH) test, at 54 days of age, 2 geese of each cage were randomly selected, and the toe web thickness between the second and third digits on the left foot was measured (in millimeters) with a constant-tension caliper and injected with 0.10 mL of dissolved phytohemagglutinin P (PHA-P) (2 mg/mL). The PHA-P is a lectin from the red kidney bean and the PHA-induced skin swelling test has been intensively considered a classical immunological technique. Then, 8, 28, and 48 h after the injection, the toe webs were measured again. The difference of the thickness of the toe web before and after injection was used as an indicator of the cellular immune response [25]. 

### 2.4. Blood Metabolites

To evaluate geese serum metabolites, blood samples were collected randomly from the wing vein of 2 male geese of each cage (8 geese per treatment), 20 h prior to slaughter. The serum samples were analyzed for total cholesterol, high-density lipoproteins (HDL), total antioxidant status (TAS), and triglycerides using an auto analyzer appurtenance (ALISON 300) and the kits were supplied by Pars Azmoun Co. Iran. The total serum antioxidant status was assayed using a Trolox (6-hydroxy-tetramethylchroman2-carboxylic acid) standard curve calibration according to Miller’s [26] method. The results were expressed in mmol/L of the Trolox equivalent antioxidant capacity. 

### 2.5. Meat Sample and Carcass Characters

At day 56 of age, 2 geese from each cage (8 geese per treatment) were selected within a BW range from 95% to 105% of the average BW per cage. The slaughtering process was conducted in the slaughter facilities of the laboratory after 12 h of the lairage period. After a 2-min bleeding, the scalding process of carcasses was carried out in hot water (60 °C) for 2 min prior to mechanical feather plucking, evisceration, and tissue sample collection. After dissecting the head, legs, and skin, the heart, gizzard, liver, and abdominal fat were removed and weighed. The warm carcasses were weighed again, and then, the back, wings, thighs, and breast were separated and weighed separately. The percentage of breast and leg muscle and other carcass parts (wing and back) were expressed with respect to the carcass weight. Carcass yield without abdominal fat was expressed with respect to the live weight. Abdominal fat and edible giblets, including the liver, heart, and gizzard, was calculated as the ratio of live body weight. A tissue sample of breast muscle obtained from the killed goose was stored at −20 °C to determine the chemical composition, lipid oxidation, total volatile base nitrogen, total phenolic content, and fatty acid composition. To evaluate the meat shelf life, breast samples were collected from both sides of the breast and they were sectioned into 2 × 2 × 2 cm, which were aerobically packaged in polypropylene bags and held in refrigerated storage at 4 °C. Physicochemical properties related to meat shelf life stability were determined at 1-, 3-, 6-, and 9-day intervals of post-mortem storage.

### 2.6. Chemical Composition Analysis of Meat

The pH of meat was determined according to Alirezalu et al.’s [27] method by homogenization of samples with distilled water (at a 1:10) using a Ultra-Turrax homogenizer (IKA, T50 Ultra-Turrax, Werke, Staufen, Germany) set at 12,000 rpm, for 2 min. The moisture, fat, and protein (Kjeldahl method) contents, and ash composition of the meat were measured in accordance to the Association of Analytical Chemists [28] methods.

### 2.7. Total Phenolic Content of Meat

The phenolic compounds of meat samples were performed following the Folin–Ciocalteu method according to Liu et al. [29]. Briefly, the sample absorbance at the 700-nm wavelength was determined using a spectrophotometer (Hitachi, Ltd., Tokyo, Japan). Results were calculated against the calibration curve, performed with standard of gallic acid in the range of 0.00 to 0.03 mg/mL. The total phenolic content was expressed as mg per 100 g of dry matter.

### 2.8. Lipid Oxidation of Meat (TBARS)

Lipid oxidation was quantified using the thiobarbituric acid reactive substances (TBARS) analysis to determine the amount of malondialdehyde aldehyde. TBARS content was determined using a spectrophotometer (JENWAY-6405, San Diego, CA, USA) set to measure the absorbance at 532 nm. The results were expressed as mg of malondialdehyde per kg of fresh meat [30]. 

### 2.9. Total Volatile Base Nitrogen of Meat (TVB-N)

The Kjeldahl method was used to calculate the total volatile base nitrogen. The TVB-N content of meat and liver samples was performed according to the method of Harold et al. [31]. Results were expressed as mg nitrogen per 100 g fresh meat.

### 2.10. Fatty Acids Composition of Meat

Duplicate fresh meat samples were minced, and their lipid content extracted in accordance with Folch et al.’s [32] method. The fatty acid composition was identified and measured by gas chromatography. The separation of fatty acid methyl esters (FAMEs) was carried out with an Agilent capillary column (30 m × 0.25 mm I.D., CPS Analitica, Milan, Italy) as reported by Pintado et al. [33]. Individual FAMEs were identified based on their retention time and tridecanoic acid (C13:0) methyl ester was added before extraction as an internal standard. The FAMEs were calculated by integration under the peak areas and expressed as mg fatty acid/100 g meat. The peroxidability index (PI) of fatty acids was determined as described in the method by Arakawa and Sagai [34].

### 2.11. Statistical Analysis

A total of 64 male geese were distributed in a completely randomized design with 2 treatments of 4 replicates. The experimental unit was the cage mean (8 geese each). Apart from the data for the performance parameters, all other data were analyzed with ANOVA using the General linear model (GLM) procedure of Statistical Analysis Software (SAS). Performance data were subjected in the repeated measurement analysis of the PROC MIXED procedure of SAS with 2 experimental diets (control and supplemented with vitamin E), and 4 rearing phase (1–14 days, 14–28 days, 28–42 days, and 42–56 days) as fixed effects. Percentage data corresponding to carcass defects were transformed to arcsine√% for analysis. The moisture, fat, protein, and ash contents were analyzed according to a repeated-measures experimental design with the MIXED procedure. The least square mean was used to determine the groups significantly different from each other. Mean differences were compared using Tukey’s tests at the *p* < 0.05 level.

## 3. Results and Discussion

### 3.1. Effect on Vitamin E Supplementation on Growth Performance

The effect of vitamin E supplementation on the growth parameters of geese from 1 to 56 days of age is shown in Table 2. In the present study, the vitamin E concentration in the control diet was an adequate level to meet the geese’s nutritional requirements suggested by NRC [35], and the supplemented diet had the same ingredients plus 120 mg/kg of extra vitamin E. 

The results showed that vitamin E supplementation (120 mg/kg diet) did not have a significant effect on the average daily feed intake (ADFI). As expected, ADFI was statistically affected by rearing phase (*p* < 0.01); the ADFI was increased linearly until phase 3 but decreased in phase 4, reflecting that in phase 3, the birds consumed more than in the other phases. The interaction between rearing phase and dietary treatment on ADFI was also significant (*p* < 0.01) in phase 3, in which birds fed diet supplemented with vitamin E had a higher feed intake than those fed the control diet. However, in phase 4, there was a substantial decrease in ADFI in the supplemented diet group, but the control group showed no significant differences.

In contrast to ADFI, the average daily gain (ADG) was significantly (*p* < 0.01) improved (54.69 vs. 46.36 g/day) with supplementation of vitamin E in the geese’s diet. Meanwhile, the rearing phase effect on ADG followed another trend. The geese’s ADG in phases 2 and 3 was statistically (*p* < 0.01) greater than in phases 1 and 4. On the other hand, the ADG in phase 4 was not statistically different (*p* > 0.05) from the value obtained in phase 1 and significantly lower (*p* < 0.01) than those reported in phases 2 and 3. Additionally, there was no phase × diet interaction in ADG between treatments. The final BW of the group that received the supplemented diet was higher than the control group (*p* < 0.05), and as expected, the geese in phase 4 were heavier than in the other phases (*p* < 0.01). There was a phase × diet interaction in BW between treatments. In all phases except for phase 1, the geese that consumed the supplemented diet were heavier than those fed with the control diet (*p* < 0.05). As the ADFI did not differ between the two dietary groups, while the ADG was significantly improved, the supplementation with vitamin E allowed a significant (*p* < 0.05) reduction of 10% in the FCR with respect to the control group. The FCR was statistically affected by the rearing phase (*p* < 0.01), and as expected linearly increased as the age of the geese increased. A significant diet × rearing phase interaction (*p* < 0.01) in FCR between treatments was observed in the final phase (phase 4), and the vitamin E group showed lower FCR with respect to the control diet (4.81 vs. 5.9). The supplementation of vitamin E in the geese’s diet statistically (*p* < 0.05) improved the EPEF (215 vs. 190), which decreased linearly as the age of the geese increased (*p* < 0.01). However, there was no phase × diet interaction in EPEF between treatments.

Some research has addressed the effect of vitamin E supplementation on the growth performance and immunity of broilers or breeders [16,17,18]; however, few previously published studies have addressed the issue of the growth rate in geese. Łukaszewicz et al. [23] fed White Kołuda geese a 0.3 mg/kg selenium- and 100 mg/kg vitamin E-supplemented diet from day 1 to 112 of age. However, the used ingredients in the experimental diets were not reported. In this study, the researchers indicated that the supplementation of the diet with 0.3 mg/kg of selenium and 100 mg/kg of vitamin E statistically improved the BW and FCR of White Kołuda geese.

Niu et al. [17], working with broilers under heat stress, fed three dietary treatments (control with 10.25 mg/kg vitamin E, control plus 100 mg/kg vitamin E, and control plus 200 mg/kg vitamin E) and did not find a significant effect on BW and FI by supplementation of vitamin E. On the contrary, the FCR was significantly influenced, with geese fed 100 mg/kg vitamin E showing lower FCR. Additionally, these authors did not report a significant interaction in the growth performance parameters, between the dietary vitamin E level and environmental temperature. In partial agreement with this, Laganá et al. [16] reported that supplementation of vitamins E and C in a poultry diet at a high temperature decreases FI and improves FCR. These authors suggested that the growth performance of broilers submitted to heat stress can be strongly influenced by the supplementation of vitamins or minerals [16]. Indeed, a beneficial effect of vitamin E supplementation on the poultry diet is highly variable and depends on the level and type of dietary fat (saturated or unsaturated), the levels of vitamin E and selenium, the presence of pro-oxidants and antioxidants in the diet [34], as well as environmental or physiological conditions. In the present study, there was a beneficial effect of vitamin E supplementation on FCR performance. It seems that this result could be due to a concomitant effect that increased nutrient absorption. This finding corroborates the ideas of [36], who found in Japanese quail, the digestibility of nutrients was increased by some minerals and vitamins (like, e.g., vitamin E), suggesting that an improvement in the efficiency of the use of nutrients might cause a decrease in FCR.

### 3.2. Effect on Vitamin E Supplementation on Blood Metabolites and Cellular Immunity

The effect of vitamin E supplementation on the serum TAS and lipid profile of geese on day 56 of age is shown in Table 3. The results showed that none of the factors of the serum profile were affected by the dietary treatment. Despite the fact that the total cholesterol and TAS numerically increased, the change was not statistically significant. Some studies evaluated the effect of dietary vitamin E supplementation on birds’ serum TAS and cholesterol [19,37,38,39,40] with mixed results. For instance, a study on broilers supplemented with vitamin E indicated that serum TAS was significantly enhanced compared to birds that received the control diet [16]. In contrast, other researchers reported that the serum TAS was not affected by dietary vitamin E supplementation in broilers [38] and laying hens [41]. In the same line, a recent study reported that the supplementation of vitamin E in Japanese quail diets reduced the serum total cholesterol and triglycerides [42]. This finding supports previous studies [37,39,40], who reported a reduction of the serum total cholesterol of birds fed a vitamin E-supplemented diet under heat stress conditions. The present study was done in normal conditions and none of the measured metabolites in the serum (HDL, cholesterol, TG, and TAS) were influenced by dietary supplementation of vitamin E. As previous studies have reported the positive effects of vitamin E in birds under stress conditions, it seems that the absence of a significant response to dietary supplementation in the present study may associated to the lack of environmental or physiological disorders.

The effect of vitamin E supplementation on the CBH test to PHA-P in geese at day 54 of age is shown in Table 3. This test, as a thymus-dependent reaction mediated by T (thymic) cells, is a rapid and simple [25] way to evaluate cellular immunity in growing birds. Koutsos et al. [43] observed that the increase of the skin thickness in the CBH test can be caused by the increased presence of macrophages and heterophils at the injection site, as well as basophils and lymphocytes. The swelling of toe webs was evaluated three times (8, 24, and 48 h) after the injection of PHA-P. There was no difference in the swelling of toe webs between dietary treatments at 8 and 28 h after injection. However, 24 h after injection, the toe web increased significantly (*p* < 0.05), 43% more in geese fed the vitamin E-supplemented diet than in geese fed the control diet. Leshchinsky and Klasing [18] conducted three trials to evaluate the relationship between the dietary level of vitamin E and the immune response of broilers. They found a dose-dependent increase in antibody production in response to sheep red blood cells and infectious bronchitis virus antigens; however, antibody production in response to *Brucella abortus* antigens and CBH response to PHA-P were not affected by vitamin E supplementation. However, the dose-dependent effect is controversial, because Boa-Amponsem et al. [44] reported that the addition of 300 mg/kg vitamin E had a lower response as compared to 10 mg/kg in rooster diets. Koutsos et al. [43] evaluated the influence of lutein on cellular immunity using the CBH test, assessing the cell response 48 h after injection. These authors indicated that lutein activated lymphocyte multiplication and reduced the production of free radicals in response to injected PHA-P. The mechanism of the main action of vitamin E in cellular immunity is not completely understood [18]. Similarly to lutein, vitamin E acts as an antioxidant. It is possible, therefore, that the observed difference in the inflammatory response to PHA-P in the present study is mainly associated with the antioxidant potential of vitamin E. 

### 3.3. Effect on Vitamin E Supplementation on Carcass Characteristics

The effect of vitamin E supplementation on the carcass parameters of geese on day 56 of age is shown in Table 4. The live and carcass weights were not affected by the dietary treatment, but the carcass yield of geese fed the vitamin E-supplemented diet was significantly (*p* < 0.05) higher (79.72% vs. 76.36 %) than those fed the control diet. The thigh, wing, and breast yield, as well as the abdominal fat, heart, gizzard, and liver percentages (% of live weight) were not affected by the dietary treatment. It has been indicated that deficiency of vitamin E causes gizzard myopathy in turkeys and ducks [45]. However, in the present study, the dietary levels of vitamin E were adequate in meeting the geese’s nutritional requirements. 

### 3.4. Effect on Vitamin E Supplementation on the Chemical Composition of Goose Meat

The effect of vitamin E supplementation on the chemical composition (moisture, fat, protein, and ash) of goose meat is shown in Table 5, indicating that at day 1, samples from the vitamin E group had significantly higher fat, protein, and ash contents and a lower moisture content than those from the control group. These results agree with the findings of Guo et al. [46], who evaluated the effect of dietary vitamin E supplementation on pork, showing an increase in the meat marbling score and intramuscular fat (IMF). During the storage period, there were no significant differences between treatments for the protein content, whereas the moisture content in all meat samples decreased significantly. However, the fat and ash contents increased significantly (*p* < 0.05) during storage.

### 3.5. Effect on Vitamin E Supplementation on pH, TBARS, TVB-N, and Phenolic Compounds

**Figure 1:**
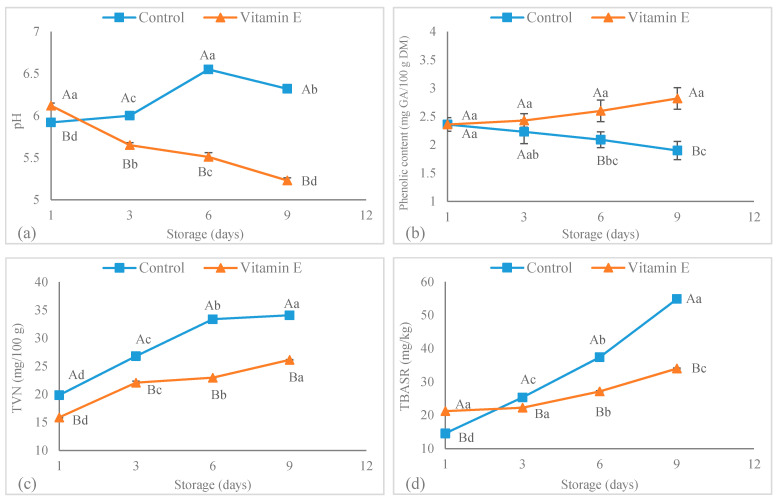
Changes in pH (**a**), phenolic content (**b**), thiobarbituric acid reactive substances= TBARS index (**c**) and total volatile base nitrogen=TVN (**d**) of goose meat supplemented with vitamin E at 4 °C during storage. ^a–d^ Quality values level within treatment during storage with different letters differ significantly (*p* < 0.05); ^A–B^ Quality values level between treatments with different letters differ significantly (*p* < 0.05).

The change in the pH values of goose meat is depicted in Figure 1a. The increment in the pH during storage has a negative effect on meat color attributes, because the yellowness (b*) and lightness (L*) of the meat decline and the meat becomes darker [47,48]. As can be observed in Figure 1a, the pH values of goose meat fed with vitamin E significantly decreased during refrigerated storage. Meanwhile, in the control samples, the pH increased. Therefore, vitamin E in goose meat had a positive effect on color attributes. Smith et al. [49] also reported a decrease in the pH of alpaca meat in comparison with the corresponding control samples (without vitamin E supplementation). The growth of lactic acid bacteria in aerobic packaging meat samples could be the reason for the pH increasing during storage [50]. Despite the fact that this finding must be interpreted with caution, it can thus suggest that some antimicrobial activity of vitamin E may be the main reason pH decreases during storage.

One of the factors most affecting meat quality and meat shelf life is lipid oxidation [51]. Meat lipid oxidation is usually associated with reactions that lead to the production of lipid peroxides and hydroperoxides, the accumulation of which can be assessed by TBARS values [52,53]. The TBARS values in all meat samples studied increased significantly during the storage period, but the rate of this increase was significantly lower in goose meat fed the vitamin E diet than in control ones (Figure 1c). This result may be explained by the fact that vitamin E has antioxidant properties. This compound can react with lipids and other free radicals by locating the lipophilic part in the cell membranes and preventing oxidation reactions [54]. The antioxidant properties of vitamin E were also reported for broiler chickens [55]. The authors showed that the meat from broilers fed with vitamin E had significantly lower TBARS than those values in control samples. It has been reported that selenium supplementation in combination with vitamin E supplementation improved the antioxidant properties of both the liver and muscle of geese at a concentration of 80 mg/kg diet [21]. Furthermore, vitamin E can increase meat color stability by delaying oxymyoglobin conversion to metmyoglobin during storage [14,56,57]. The antioxidant properties of vitamin E and plant extracts have been confirmed in the meat of different animal species, such as Dohne Merino lambs [58], pigs [59], and meat products [60]. One of the most important freshness indicators in meat products is the TVB-N value [61]. Enzymatic degradation and the growth of microorganisms during storage can lead to protein decomposition and the production of alkaline nitrogen components that can be expressed as TVB-N values. High values of this parameter significantly affect the shelf life and quality of meat and meat products [24,62]. The TVB-N values in all goose meat samples studied increased continuously during storage, but the rate of this increase was significantly higher in control samples than those values in goose meat from the vitamin E treatment (Figure 1b). The high antioxidant capacity of vitamin E along with a possible antimicrobial effect that leads to lower microbial populations in meat samples could explain the lower TVB-N values in samples with vitamin E. Phenolic compounds, with antimicrobial and antioxidant properties, are known as important functional ingredients [63,64,65]. The changes in the phenolic compounds of goose meat are depicted in Figure 1d. During storage, the phenolic compounds in the control samples declined continuously. This observed decrease could be attributed to its use to counter free radicals caused by oxidation reactions. In contrast, the phenolic compounds in the samples treated with vitamin E were significantly higher than control ones.

### 3.6. Effect on Vitamin E Supplementation on the Meat Fatty Acid Composition

The effect of vitamin E supplementation on the fatty acid composition of goose meat is shown in Table 6. The fatty acid composition in meat is associated to the diet’s fatty acid consumption, especially in monogastric animals because they do not substantially modify the fat consumed during the digestion of feed, and deposit this fat in tissues with little or no modification [66]. Furthermore, from a nutritional point of view, the PUFA content in meat can significantly affect human health, and additionally, compounds from feeds contribute to the final flavor of fresh meat [67,68]. 

Among the fatty acids, the most abundant were in the following order: Palmitic acid (C16:0) followed by linoleic acid (18:2n-6), oleic acid (18:1n9), and stearic acid (18:0) in goose meat from both treatments. The PUFA content in goose meat fed vitamin E was significantly (*p* < 0.05) higher than control samples, because of the linolenic acid content (1072.47 vs. 802.66 mg fatty acid/100 g meat for vitamin E and control samples, respectively). The peroxidability index was calculated from the composition ratio and the reactivity of each polyunsaturated fatty acid. The peroxidability index was affected by the dietary treatments, and goose meat supplemented with vitamin E had a higher PI compared to the control group. Similar results were also reported in lamb meat [69] and pork [46]. It has been suggested that the antioxidant properties of vitamin E in terms of preventing lipid oxidation and consequently decreasing free radical formation in muscle could protect unsaturated fatty acids as the PUFA [56,70,71]. When the geese’s diet was supplemented with vitamin E, increases in C14:0 (myristic acid), C16:0 (palmitic acid), C16:1 (palmitoleic acid), and C18:1 (oleic acid) were observed, as shown in Table 6, because of the greater content in the intramuscular fat of the vitamin E group. To meet market demands (higher MUFA and PUFA in meat), the modification of the fatty acid composition of goose meat, using vitamin E in the diets of geese, could be a successful strategy to achieve it. 

## 4. Conclusions

Dietary supplementation with vitamin E improved the growth performance, carcass yield, and immune response compared to the control group. From a nutritional and health point of view, goose meat supplemented with vitamin E improved in terms of significant increments of polyunsaturated fatty acids, such as linolenic acid. Moreover, this study showed that vitamin E supplementation led to goose meat with a greater shelf life compared to the control, which would be appreciated by retailers and meat processors. Further studies need to be done to establish the optimum doses of vitamin E to be used in geese rearing.

## Figures and Tables

**Table 1:** Composition and calculated nutritional levels of the control diet.

Ingredients (%)	1 to 4 Weeks	5 to 8 Weeks
Corn	43	55
Wheat	24	26
Soybean meal	30	16
Di-calcium phosphate	1	1
Oyster shell	1	1
Common salt	0.1	0.1
Mineral premix ^1^	0.25	0.5
Vitamin premix ^2^	0.25	0.5
L-Lysine	0.2	0.2
DL-Methionine	0.2	0.2
Calculated nutritional composition		
AMEn (kcal/kg)	2922	2911
Crude protein (g/kg)	202.9	145.2
Crude fiber (g/kg)	43.0	44.6
Crude fat (g/kg)	22.4	26.7
Total lysine (g/kg)	13.5	9.4
Total methionine + cysteine (g/kg)	7.6	6.2
Total threonine (g/kg)	8.7	6.4
Calcium (g/kg)	6.8	6.6
Available phosphorus (g/kg)	4.0	3.7
Sodium (g/kg)	1.1	1.1
Selenium (mg/kg diet)	0.17	0.17
Vitamin E (mg/kg diet)	11.80	14.05

^1^ The mineral contained per kg of diet: Manganese oxide, 24 g; Iron sulfate, 16 g; Copper sulfate, 3 g; Calcium iodate, 0.2 g; Cobalt, 0.08 g. ^2^ The vitamin contained per kg of diet: Vit A (retinol), 36 × 10^7^ IU; Vit D_3_ (cholecalciferol), 8 × 10^5^ IU; K_3_ (menadione), 7 g; B_1_ (thiamine), 0.7 g; B_2_ (riboflavin), 2.64 g; Nicotinic acid, 4 g; Pantothenic acid, 12 g; Peridoxin, 1.2 g; Biotin, 0.04 g; Folic acid, 0.4 g; Cobalamin, 0.006 g.

**Table 2:** Influence of dietary supplementation of vitamin E on the growth performance of 1- to 56-day-old male geese.

	ADFI (g/day)	ADG (g/day)	BW (g)	FCR	EPEF
Effect of diet					
Control	147.90	46.36 ^b^	1739.51 ^b^	3.31 ^a^	190.59 ^b^
Vitamin E	160.90	54.69 ^a^	1955.30 ^a^	2.96 ^b^	215.76 ^a^
SEM	6.53	1.62	57.78	0.07	6.85
Effect of phase					
Phase 1 (days 1–14)	43.91 ^d^	34.82 ^b^	579.60 ^d^	1.26 ^d^	329.18 ^a^
Phase 2 (days 14–28)	177.57 ^c^	66.19 ^a^	1506.33 ^c^	2.75 ^c^	203.43 ^b^
Phase 3 (days 28–42)	206.36 ^a^	65.32 ^a^	2420.83 ^b^	3.17 ^b^	182.40 ^b^
Phase 4 (days 42–56)	189.74 ^b^	35.77 ^b^	2882.86 ^a^	5.36 ^a^	97.69 ^c^
SEM	5.53	2.50	51.23	0.11	12.06
Effect of diet × phase					
Control × Phase 1	43.08 ^d^	34.86	580.09 ^f^	1.23 ^e^	335.67
Control × Phase 2	166.51 ^c^	58.04	1392.70 ^e^	2.91 ^cd^	175.19
Control × Phase 3	190.65 ^b^	60.24	2236.14 ^c^	3.17 ^c^	168.46
Control × Phase 4	191.35 ^b^	32.30	2749.11 ^b^	5.90 ^a^	83.04
Vitamin E × Phase 1	44.75 ^d^	34.79	579.11 ^f^	1.29 ^e^	322.69
Vitamin E × Phase 2	188.62 ^bc^	74.34	1619.96 ^d^	2.60 ^d^	231.66
Vitamin E × Phase 3	222.07 ^a^	70.39	2605.51 ^b^	3.16 ^c^	196.33
Vitamin E × Phase 4	188.14 ^bc^	39.24	3016.61 ^a^	4.81 ^b^	112.34
SEM	7.82	3.54	72.45	0.16	17.05
*F*(df1, df2)=x, *p*-value					
Diet	*F*(1, 6) = 1.98, *p* < 0.2094	*F*(1, 6) = 13.16, *p* < 0.0110	*F*(1, 6)= 6.97, *p* < 0.0385	*F*(1, 6) = 10.29, *p* < 0.0184	*F*(1, 6) = 6.73, *p* < 0.0410
Phase	*F*(3, 18) = 394.1, *p* < 0.0001	*F*(3, 18) = 47.3, *p* < 0.0001	*F*(3, 18) = 492.9, *p* < 0.0001	*F*(3, 18) = 217.4, *p* < 0.0001	*F*(3, 18) = 71.4, *p* < 0.0001
Diet × Phase	*F*(3, 18) = 7.54, *p* < 0.0018	*F*(3, 18) = 1.72, *p* < 0.1996	*F*(3, 18) = 4.02, *p* < 0.0237	*F*(3, 18) = 4.74, *p* < 0.0132	*F*(3, 18) = 1.18, *p* < 0.3456

Means within each column with no common superscript differ significantly at *p* < 0.05; BW: body weight, ADG: average daily gain, ADFI: average daily feed intake, FCR: feed conversion ratio, EPEF: European poultry efficiency factor.

**Table 3:** Effect of dietary vitamin E supplementation on serum TAS ^1^, cholesterol, triglyceride, and response to the CBH ^2^ test of male geese.

Serum Metabolites ^3^				
Treatment	HDL (mg/dL)	Cholesterol (mg/dL)	TG (mg/dL)	TAS (mmol)
Control	72.383	196.000	57.67	0.816
Vitamin E	76.683	185.333	64.33	0.893
SEM	3.38	6.23	9.57	0.0626
*F*(df1, df2) = x, *p*-Value	*F*(1, 6) = 0.81, *p* < 0.4031	*F*(1, 6) = 1.46, *p* < 0.2718	*F*(1, 6) = 0.24, *p* < 0.6401	*F*(1, 6) = 0.75, *p* < 0.4201
**Response to CBH test**				
	**Toe web swelling after different times (mm)**			
		8 h	24 h	48 h
Control		0.1487	0.1425 ^b^	0.1437
Vitamin E		0.1650	0.2037 ^a^	0.1850
SEM		0.0208	0.0132	0.0250
*F*(df1, df2) = x, *p*-Value		*F*(1, 6) = 0.30, *p* < 0.6012	*F*(1, 6) = 10.73, *p* < 0.0169	*F*(1, 6) = 1.36, *p* < 0.2871

Means within each column with no common superscript differ significantly at *p* < 0.05; ^1^ TAS: Total antioxidant status; ^2^ CBH: Cutaneous basophil hypersensitivity; ^3^ HDL: High density lipoprotein, TG: Triglyceride.

**Table 4:** Effect of vitamin E supplementation on carcass parameters of 56-day-old male geese.

Parameters	Control	Vitamin E	SEM	*F*(df1, df2) = x, *p*-Value
Carcass weight (g)	2198.3	2493.8	102.2	*F*(1, 6) = 4.17, *p* < 0.087
Liver (g)	60.00	75.00	8.53	*F*(1, 6) = 1.54, *p* < 0.260
Gizzard (g)	111.66	106.25	6.87	*F*(1, 6) = 0.31, *p* < 0.597
Heart (g)	25.00	22.50	1.02	*F*(1, 6) = 3.00, *p* < 0.134
Carcass yield (%)	76.32 ^b^	79.72 ^a^	0.77	*F*(1, 6) = 9.63, *p* < 0.021
Breast (%)	18.07	17.46	0.861	*F*(1, 6) = 0.25, *p* < 0.633
Back (%)	26.46	26.60	0.92	*F*(1, 6) = 0.01, *p* < 0.525
Thigh (%)	21.33	22.47	1.42	*F*(1, 6) = 0.32, *p* < 0.590
Wing (%)	14.86	14.36	0.52	*F*(1, 6) = 0.45, *p* < 0.526
Liver (%)	2.05	2.38	0.219	*F*(1, 6) = 1.14, *p* < 0.325
Heart (%)	0.85	0.72	0.049	*F*(1, 6) = 3.67, *p* < 0.103
Gizzard (%)	3.81	3.39	0.180	*F*(1, 6) = 2.69, *p* < 0.152
Abdominal fat (%)	2.28	2.45	0.373	*F*(1, 6) = 0.12, *p* < 0.739

Means within each row with no common superscript differ significantly at *p* < 0.05.

**Table 5:** Effect of vitamin E supplementation on the chemical composition (%) of goose meat during storage at 4 °C.

Parameters	Treatment	Storage Time (Day)				*F*(df1, df2) = x, *p* < 0.05
		1	3	6	9	
Moisture	68.64 ± 0.05 ^Aa^	67.52 ± 0.01 ^Ab^	66.94 ± 0.05 ^Ac^	64.44 ± 0.04 ^Ad^	68.64 ± 0.05 ^Aa^	*F*(3, 12) = 6934.73, *p* < 0.0001
	67.09 ± 0.02 ^Ba^	66.75 ± 0.05 ^Bb^	65.79 ± 0.02 ^Bc^	63.82 ± 0.04 ^Bd^	67.09 ± 0.02 ^Ba^	
Fat	5.53 ± 0.04 ^Bd^	6.28 ± 0.02 ^Bc^	6.59 ± 0.004 ^Bb^	6.95 ± 0.017 ^Ba^	5.53 ± 0.04 ^Bd^	*F*(3, 12) = 397.72, *p* < 0.0001
	6.62 ± 0.11 ^Ac^	7.77 ± 0.02 ^Ab^	7.87 ± 0.007 ^Ab^	8.41 ± 0.005 ^Aa^	6.62 ± 0.11 ^Ac^	
Protein	20.58 ± 0.17 ^B^	20.58 ± 0.11 ^B^	20.62 ± 0.16 ^B^	20.93 ± 0.17 ^B^	20.58 ± 0.17 ^B^	*F*(3, 12) = 1.76, *p* < 0.2082
	22.53 ± 0.27 ^A^	22.32 ± 0.04 ^A^	22.61 ± 0.13 ^A^	22.77 ± 0.26 ^A^	22.53 ± 0.27 ^A^	
Ash	1.86 ± 0.005 ^Bd^	2.01 ± 0.0003 ^Ac^	2.11 ± 0.002 ^Ab^	2.41 ± 0.006 ^Aa^	1.86 ± 0.005 ^Bd^	*F*(3, 12) = 858.22, *p* < 0.0001
	1.93 ± 0.012 ^Ad^	1.95 ± 0.007 ^Bc^	1.98 ± 0.003 ^Bb^	2.07 ± 0.004 ^Ba^	1.93 ± 0.012 ^Ad^	

^a–d^ Chemical values level within each row during storage with different letters differ significantly (*p* < 0.05); ^A–D^ Chemical values level within each column between treatments with different letters differ significantly (*p* < 0.05).

**Table 6:** Effect of vitamin E supplementation on the fatty acid composition (mg fatty acid/100 g meat) of goose meat.

Fatty Acids	Control	Vitamin E	SEM	*F*(df1, df2) = x, *p* < 0.05
C9:0 (Pelargonic acid)	21.75	36.96	8.21	*F*(1, 4) = 1.72, *p* < 0.260
C10:0 (Capric acid)	223.10	331.82	62.89	*F*(1, 4) = 1.50, *p* < 0.288
C11:0 (Undecanoic acid)	18.78	29.95	6.13	*F*(1, 4) = 1.66, *p* < 0.267
C12:0 (Lauric acid)	173.32	272.30	37.52	*F*(1, 4) = 3.49, *p* < 0.135
C13:0 (Tridecylic acid)	21.75	26.06	5.79	*F*(1, 4) = 0.28, *p* < 0.625
C14:0 (Myristic acid)	16.48 ^b^	49.79 ^a^	1.24	*F*(1, 4) = 356.04, *p* < 0.0001
C16:0 (Palmitic acid)	1122.94 ^b^	1312.88 ^a^	13.85	*F*(1, 4) = 94.17, *p* < 0.0006
C18:0 (Stearic acid)	972.00	900.92	34.35	*F*(1, 4) = 2.15, *p* < 0.216
C20:0 (Arachidic acid)	0.11	0.17	0.029	*F*(1, 4) = 0.84, *p* < 0.342
**SFA**	2570.23 ^b^	2960.85 ^a^	6.38	*F*(1, 4) = 1874.12, *p* < 0.0001
C14:1 (Myristoleic acid)	19.77 ^a^	10.01 ^b^	4.03	*F*(1, 4) = 12.00, *p* < 0.025
C16:1 (Palmitoleic acid)	113.68 ^b^	153.27 ^a^	9.05	*F*(1, 4) = 9.57, *p* < 0.036
C18:1 (Oleic acid)	1717.4 ^b^	2101.80 ^a^	76.87	*F*(1, 4) = 12.53, *p* < 0.024
C20:1 (Gadoleic acid)	0.08	0.11	0.027	*F*(1, 4) = 0.88, *p* < 0.071
**MUFA**	1850.93 ^b^	2255.19 ^a^	70.84	*F*(1, 4) = 16.32, *p* < 0.016
C18:2 (Linoleic acid)	802.66 ^b^	1072.47 ^a^	16.06	*F*(1, 4) = 141.19, *p* < 0.0003
C18:3 (α-Linolenic acid)	1.33	1.34	0.16	*F*(1, 4) = 0.76, *p* < 0.214
C20:4 (Arachidonic acid)	677.10	735.60	16.16	*F*(1, 4) = 6.56, *p* < 0.062
C22:6 (Docosahexaenoic acid)	36.24	64.57	13.08	*F*(1, 4) = 2.35, *p* < 0.200
**PUFA**	1517.33 ^b^	1873.98 ^a^	10.86	F(1, 4) = 539.89, *p* < 0.0001
**PUFA/SFA**	0.59 ^b^	0.63 ^a^	0.002	*F*(1, 4) = 169.00, *p* < 0.0002
**n3**	37.58 ^a^	65.91 ^a^	13.07	*F*(1, 4) = 2.35, *p* < 0.199
**n6**	1479.78 ^b^	1808.07 ^a^	4.65	*F*(1, 4) = 2486.17, *p* < 0.0001
**PI**	3850.00 ^b^	4590.50 ^a^	142.10	*F*(1, 4) = 13.61, *p* < 0.021

^a–b^ Fatty acids values level within each row with different letters differ significantly (*p* < 0.05). SFA: Saturated fatty acid; MUFA: Monounsaturated fatty acid; PUFA: Polyunsaturated fatty acid; PI: Peroxidability index.
